# Supplementary material for: Enhancement of red blood cell transfusion compatibility using CRISPR‐mediated erythroblast gene editing
Source: EMBO Mol Med. 2018 Apr 26;10(6):e8454. doi: 10.15252/emmm.201708454 (PMC5991592; doi:10.15252/emmm.201708454)
Supplement: Supplementary file 1 — Expanded View Figures PDF [file EMMM-10-e8454-s001.pdf]

Expanded View Figures

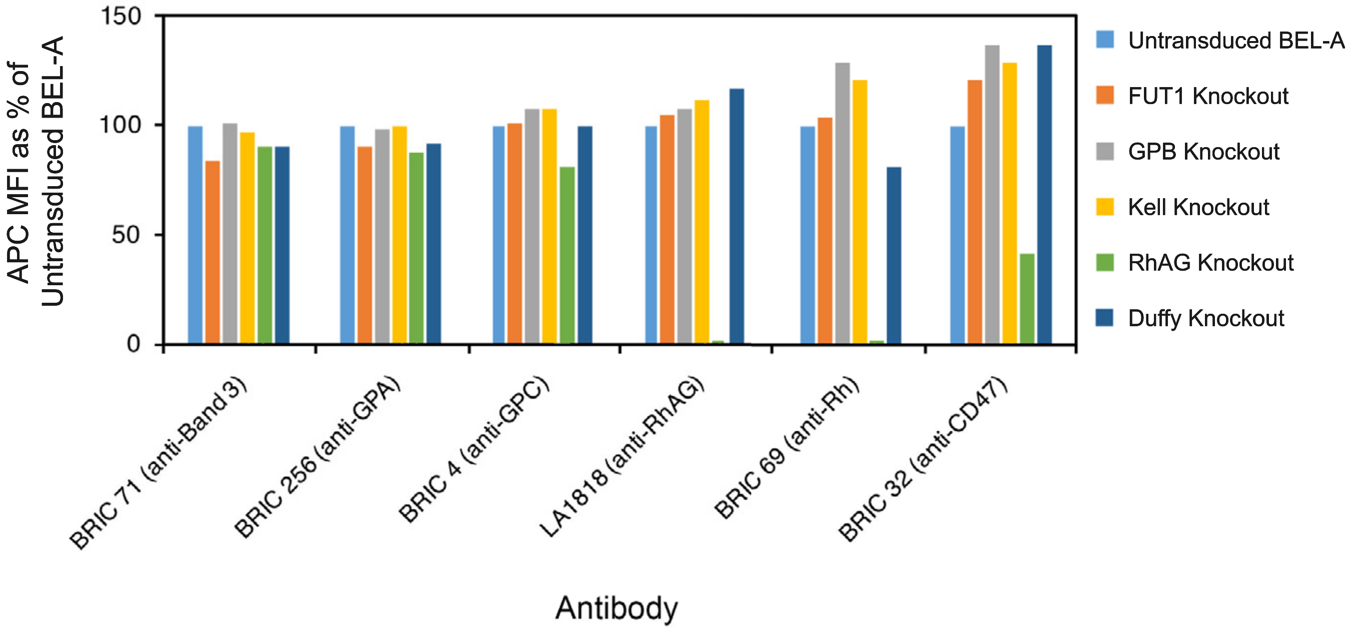

Figure EV1. Flow cytometric analysis of major erythrocyte membrane proteins in individual blood group knockout BEL-A reticulocytes.

No unexpected alterations in expression of band 3, GPA, GPC, RhAG or Rh proteins compared to untransduced BEL-A controls were observed. As expected, CD47 expression was reduced in the RhAG knockout line due to disruption of the Rh subcomplex.

 © 2018 The Authors

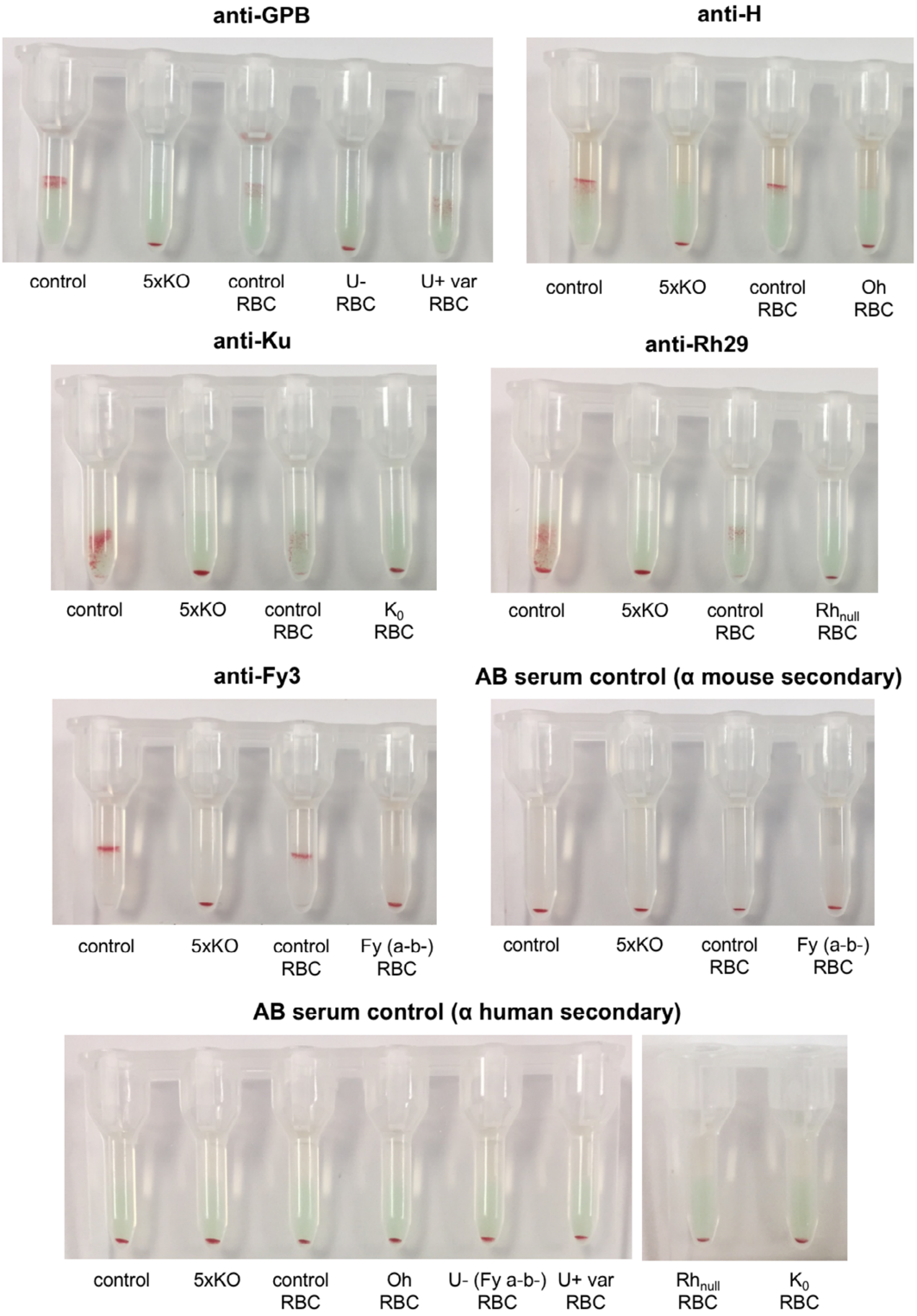

Figure EV2. Extended serological analysis of 5× KO BEL-A reticulocytes.

Gel card indirect antiglobulin tests support the absence of GPB, H antigen, Kell, Rh and Duffy in 5× KO cells using anti-U, anti-H, anti-Ku, anti-Rh29 and anti-Fy3, respectively. Cellular controls included unedited BEL-A reticulocytes, positive control RBCs and negative control RBCs. AB serum controls were performed with both anti-mouse and anti-human secondary antibodies.

© 2018 The Authors

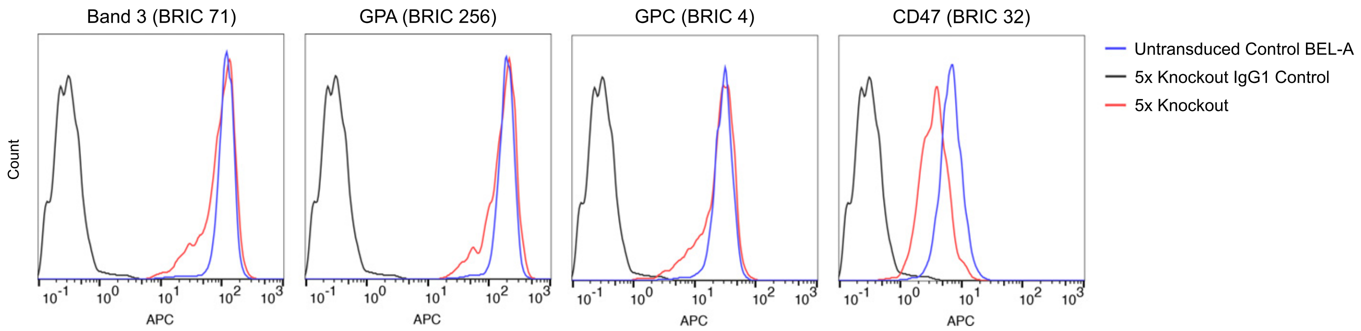

Figure EV3. Flow cytometric analysis of major erythrocyte membrane proteins in 5× knockout BEL-A reticulocytes.

No alteration in levels of band 3, GPA or GPC confirms the absence of gross membrane disruption. As expected, CD47 expression was reduced due to disruption of the Rh subcomplex.

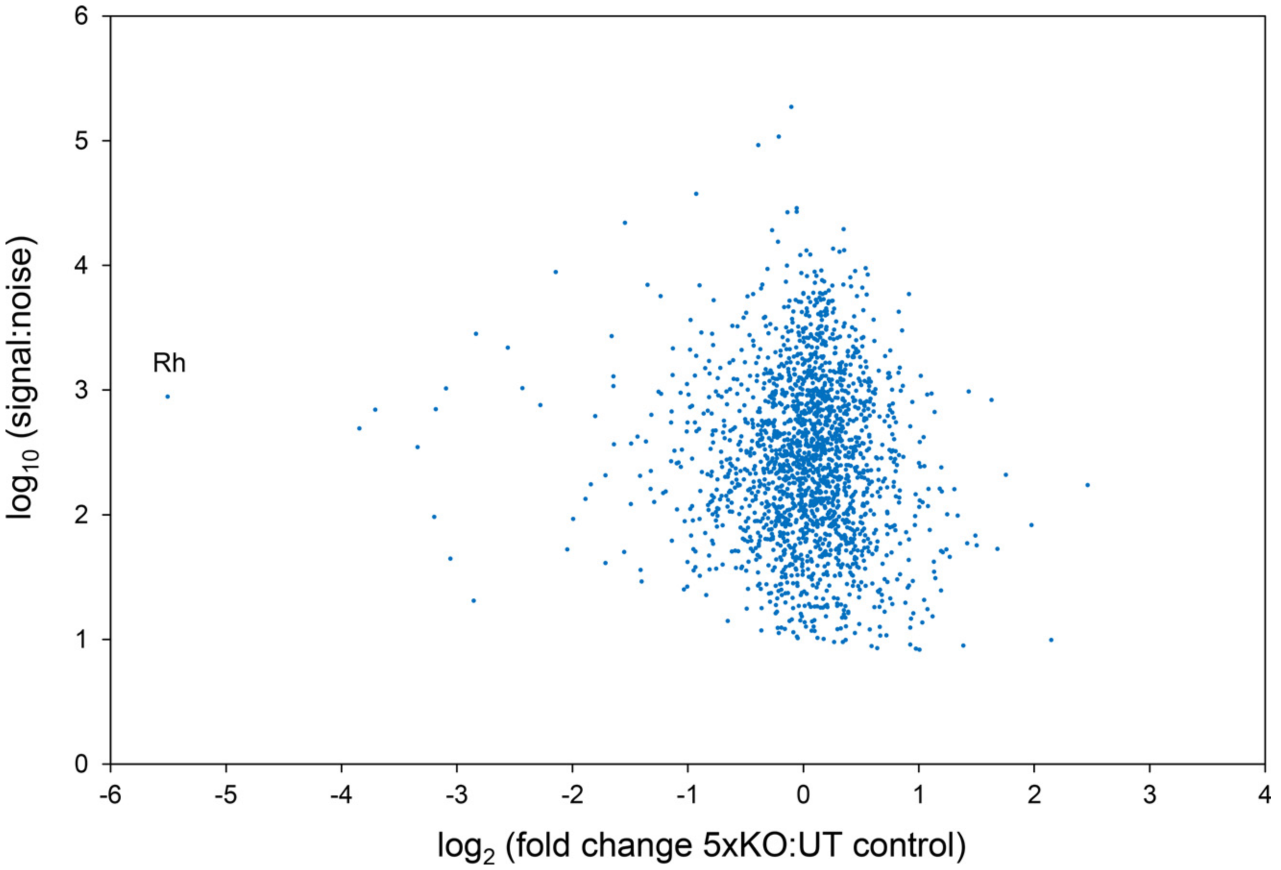

Figure EV4. Quantitative proteomics of 5× KO and untransduced BEL-A reticulocytes.

Scatter plot depicting relative protein abundance of all proteins detected in reticulocytes derived from 5× KO compared to control BEL-A cells as identified by TMT labelling and mass spectrometry. Log_2 fold ratios are based on the mean of two technical replicates. Data were filtered using a FDR of 1% with exclusion of proteins for which only a single peptide was detected. The full proteomic data set is included in Dataset EV1.

© 2018 The Authors
