# Supplementary material for: Enhancement of red blood cell transfusion compatibility using CRISPR‐mediated erythroblast gene editing
Source: EMBO Mol Med. 2018 Apr 26;10(6):e8454. doi: 10.15252/emmm.201708454 (PMC5991592; doi:10.15252/emmm.201708454)
Supplement: Supplementary file 2 — Table EV1 [file EMMM-10-e8454-s002.docx]

| **Gene** | **Chromosome** | **Amino Acid Change** | **Mutation** |
| --- | --- | --- | --- |
| *RHAG* | 6 | RHAG:NM_000324:exon2:c.287_291del:p.I96fs  RHAG:NM_000324:exon2:c.193_296del:p.F65fs | Frameshift deletion  Frameshift deletion |
| *KEL* | 7 | KEL:NM_000420:exon3:c.82_142del:p.S28fs  KEL:NM_000420:exon3:c.82_* | Frameshift deletion  Deletion* |
| *GYPB* | 4 | GYPB:NM_002100:exon3:c.G161A:p.R54H,GYPB:NM_001304382:exon4:c.G83A:p.R28H  GYPB:NM_002100:exon3:c.A166T:p.T56S,GYPB:NM_001304382:exon4:c.A88T:p.T30S  GYPB:NM_002100:exon3:c.T170A:p.V57E,GYPB:NM_001304382:exon4:c.T92A:p.V31E | Missense SNV  Missense SNV  Missense SNV |
| *FUT1* | 19 | FUT1:NM_000148:exon4:c.T640G:p.F214V  FUT1:NM_000148:exon4:c.642delT:p.F214fs  FUT1:NM_000148:exon4:c.642dupT:p.V215fs | Missense SNV  Frameshift deletion  Frameshift insertion |
| *ACKR1* | 1 | ACKR1:NM_001122951:exon1:c.212_220del:p.71_74del,ACKR1:NM_002036:exon2:c.206_214del:p.69_72del  ACKR1:NM_001122951:exon1:c.202_222del:p.68_74del,ACKR1:NM_002036:exon2:c.196_216del:p.66_72del | Non-frameshift deletion  Non-frameshift deletion |

**Table EV1. On-target CRISPR-mediated mutations in 5x KO BEL-A cells.**Biallelic mutations in targeted genes of 5x KO BEL-A cells were identified from whole genome sequencing data using GATK software. *In one allele of *KEL* gene the deletion end point could not be accurately called from available sequence fragments using GATK software, however absence of wild type sequence was confirmed.
