# Supplementary material for: Enhancement of red blood cell transfusion compatibility using CRISPR‐mediated erythroblast gene editing
Source: EMBO Mol Med. 2018 Apr 26;10(6):e8454. doi: 10.15252/emmm.201708454 (PMC5991592; doi:10.15252/emmm.201708454)
Supplement: Supplementary file 3 — Table EV2 [file EMMM-10-e8454-s003.docx]

| Gene Target | gRNA Sequence |
| --- | --- |
| *FUT1* | GACCGCCCGCGCACCTTTGT |
| *ACKR1* | TGCTGCTAGCTAGGATACCC |
| *KEL* | GATAGCTGTCAGCACCCGCC |
| *GYPB* | GTCCATCGTTTCACTGTACC |
| *RHAG* | CCAGTGGGGCACTATTGTAC |

**Table EV2.** **gRNA sequences**. Previously published pre-validated gRNA sequences utilized in this study ([Sanjana et al, 2014](#_ENREF_29)).
